# A new threat to bees? Entomopathogenic nematodes used in biological pest control cause rapid mortality in *Bombus terrestris*

**DOI:** 10.7717/peerj.1413

**Published:** 2015-11-19

**Authors:** Alexandrea Dutka, Alison McNulty, Sally M. Williamson

**Affiliations:** Natural Sciences and Psychology, Liverpool John Moores University, Liverpool, United Kingdom

**Keywords:** Bees, Biological pest control, Entomopathogenic nematodes, Pollinator health

## Abstract

There is currently a great deal of concern about population declines in pollinating insects. Many potential threats have been identified which may adversely affect the behaviour and health of both honey bees and bumble bees: these include pesticide exposure, and parasites and pathogens. Whether biological pest control agents adversely affect bees has been much less well studied: it is generally assumed that biological agents are safer for wildlife than chemical pesticides. The aim of this study was to test whether entomopathogenic nematodes sold as biological pest control products could potentially have adverse effects on the bumble bee *Bombus terrestris*. One product was a broad spectrum pest control agent containing both *Heterorhabditis sp*. and *Steinernema sp.*, the other product was specifically for weevil control and contained only *Steinernema kraussei*. Both nematode products caused ≥80% mortality within the 96 h test period when bees were exposed to soil containing entomopathogenic nematodes at the recommended field concentration of 50 nematodes per cm^2^ soil. Of particular concern is the fact that nematodes from the broad spectrum product could proliferate in the carcasses of dead bees, and therefore potentially infect a whole bee colony or spread to the wider environment.

## Background

In recent years there has been much concern about threats to pollinating insects such as bees. The exact extent of bee decline, particularly for wild pollinators, is unknown; and the cause of bee decline is most likely to be multifactorial. Along with habitat loss, major threats to bees which have been subject to scientific study include pesticides and pathogens.

The effects of pesticides on both honey bees and bumble bees have been extensively researched, with adverse behavioural effects of neonicotinoids being observed in the laboratory (Williamson, Willis & Wright, 2014; Williamson & Wright, 2013), and semi-field and field studies reporting similar adverse effects at a colony level (Feltham, Park & Goulson, 2014; Gill, Ramos-Rodriguez & Raine, 2012; Goulson, 2015). Many studies of bee pathogens have focused on the honeybee *Apis mellifera*, as disease processes are a possible contributing factor to colony collapse disorder, the phenomenon of increased bee colony losses reported by commercial apiarists (Cornman et al., 2012). *Varroa destructor*, a parasitic mite, is a major threat to honeybee health, and also acts as a vector for viral pathogens such as deformed wing virus (Rosenkranz, Aumeier & Ziegelmann, 2010). The microsporidian parasite *Nosema ceranae* has also been identified as a potential threat to honey bee health, with pesticide exposed bees becoming more susceptible to this pathogen (Pettis et al., 2012). Wild pollinators can also be affected by some of the same pathogens as honeybees, including *N. ceranae* and deformed wing virus (Arbulo et al., 2015; Genersch et al., 2006).

Much less attention has been given to potential adverse effects of biological pest control agents on bees, and it is generally assumed that integrated pest management strategies which employ biological pest control agents are safer for bees and other wildlife than chemical pesticides (Furlan & Kreutzweiser, 2015). A few studies which have directly tested biological pest control products on bees have reported that certain biological agents, such as *Bacillus thuringiensis*, and a recombinant protein derived from spider venom toxin, are relatively safe for bees (Mommaerts, Jans & Smagghe, 2010; Nakasu et al., 2014). However, biological agents are unpredictable: deliberately introduced predators and pathogens may also have off-target effects, and potentially harm wildlife (Coote, 2003; Kaiser & Heimpel, 2015). Many biological pest control agents remain less tightly regulated than chemical pesticides, and can be bought from online retailers for unrestricted use in gardens or in agriculture. Among these products, entomopathogenic nematodes are widely used for the control of a broad range insect pests (Dillman & Sternberg, 2012). Entomopathogenic nematodes used in pest control often exhibit a symbiotic relationship with certain bacteria: on entering the host insect, pathogenic bacteria are released by the parasite, and it is bacterial infection which causes the insect’s death (Dillman & Sternberg, 2012). The bacterial symbiont is *Photorhabdus* in nematodes of the genus *Heterorhabditis*, and *Xenorhabdus* in nematodes of the genus *Steinernema*. The bacterial symbionts must perform 3 separate tasks to allow successful proliferation of the nematodes within the insect host: these are to overcome insect immune defences and cause septicaemia and death; to break down the tissues of the dead insects to release nutrients for the nematodes to proliferate; then to successfully recolonise the infective juvenile nematodes which will then be released (Goodrich-Blair & Clarke, 2007). All these tasks must be successfully achieved to allow nematode proliferation in an insect host, though only the first is necessary to cause insect death of a non-viable host insect (Nielsen-LeRoux et al., 2012).

The aim of this investigation was to determine whether entomopathogenic nematodes (*Steinernema spp*. and *Heterorhabditis sp.*) marketed commercially as biological pest control agents for use in organic farming, and advertised as being safe for wildlife, had any detrimental effects on individual *Bombus terrestris* when applied to soil at the manufacturer’s recommended concentration and below.

## Materials and Methods

Bumblebees *Bombus terrestris* were obtained as commercially reared colonies from Koppert. Bees were maintained within the hive, and were not permitted free flight outdoors, to avoid any incidental pathogen exposure. 2 colonies were used during the course of the experiments described, which were performed from June to July 2015.

Entomopathogenic nematodes were obtained as commercially available mixtures from the Nemasys range marketed by BASF. The products used were “Grow Your Own” (abbreviated to GYO in this article) consisting of a mixture of *Heterorhabditis sp.* and *Steinernema spp.*, and “Vine Weevil Killer” (abbreviated to VW in this article) containing *Steinernema kraussei*. The exact composition and species mixture contained in the GYO product was proprietory and not disclosed by the manufacturer. 2 containers of each nematode product, from 2 separate batches, were used during the course of the study.

Individual bees were captured at the hive entrance and cold anaesthetised to facilitate transfer into the treatment boxes. The treatment boxes were 0.7 litre plastic boxes fitted with feeding tubes containing 50% sucrose solution. Each box contained a 2 cm depth of sterile soil. Each bee was housed individually throughout the nematode exposure treatment. Nematodes were rehydrated and diluted according to the manufacturer’s directions, and were applied to the soil in the treatment boxes at the manufacturer’s recommended concentration of 50 nematodes per cm^2^, and at the lower concentrations of 25 nematodes per cm^2^ and 10 nematodes per cm^2^. In the controls, the soil was treated with an equivalent volume of distilled water. Bee mortality was measured 24, 48 and 72 and 96 h after nematode exposure.

The duration of the experiments described here was 4 weeks in total, with the experimental procedure being repeated on different individual bees each week. One bee colony and batch of nematode products was used for the first 2 weeks, and a second colony and batch of each nematode product being used in the second 2 weeks. Each week, 5 individual bees were included in each treatment group; therefore 20 individual bees, 10 from each colony, were exposed to each treatment in total.

Carcasses of bees which died during the nematode exposure treatment were transferred to white traps (White, 1927) to allow the collection of any nematodes which proliferated in the infected bee carcass. Any nematodes proliferating in the bee carcasses were quantified after 4–5 weeks. White traps which had become dry, or which had become overgrown with mould, were not included. Nematode counts were performed by counting the number of individual nematodes in 3 × 50 µl aliquots of culture liquid, then adjusting this for the total volume of culture liquid to estimate the total number of nematodes produced from each individual infected bee. Control bee carcasses were also collected and transferred to White traps, though these were fewer in number as there was little mortality during the experimental procedure: however, some control bees were maintained within their boxes until they died, which was usually after 7–10 days, specifically for this purpose, providing White trap data for 10 individual control bees from each hive.

Data was analysed using SPSS; a Kaplan–Meier survival analysis was performed on the mortality data, and a Kruskall–Wallis test was performed on the white trap data of recovered nematodes.

## Results

### Mortality of *Bombus terrestris* exposed to entomopathogenic nematodes

**Figure 1:**
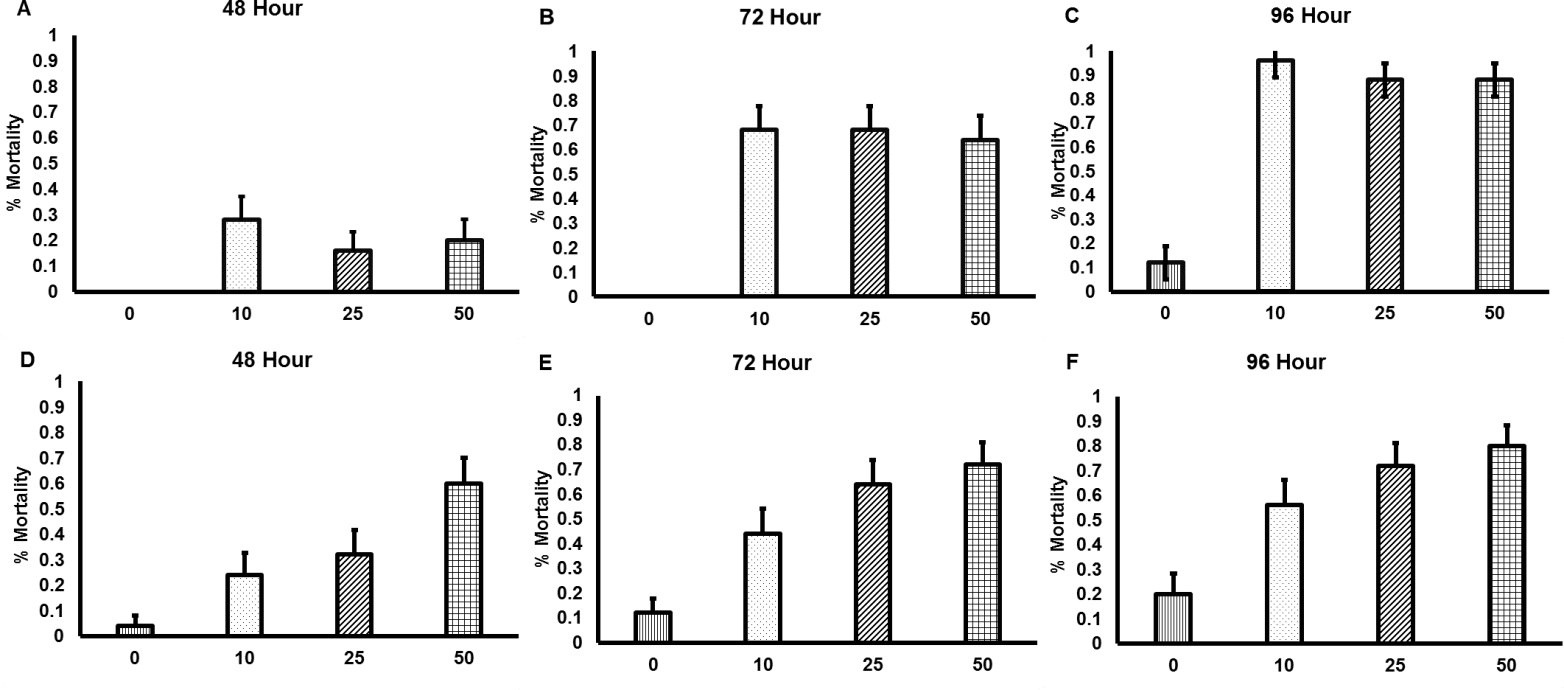
Mortality of *Bombus terrestris* exposed to entomopathogenic nematodes. Significant levels of mortality were observed in bees exposed to both the GYO product containing *Heterorhabditis sp.* and *Steinernema sp.* (A–C), and the VW product containing *Steinernema kraussei* (D–F). Mortality was recorded after 24, 48, 72 and 96 h of nematode exposure, though no mortality was observed after 24 h. Nematodes were applied to soil at the following concentrations: control (no nematodes; vertical striped bars; *n* = 20 control bees for each nematode product tested), 10 nematodes per cm^2^ (dotted bars; *n* = 20 bees for each nematode product tested), 25 nematodes per cm^2^ (diagonal striped bars bars; *n* = 20 bees for each nematode product tested), and 50 nematodes per cm^2^ (checkered bars; *n* = 20 bees for each nematode product tested). Bar charts show mean (±SE) percentage mortality.

Both commercially available nematode products rapidly killed bees, with the first incidences of mortality being observed after 48 h of nematode exposure (Fig. 1). All concentrations of the GYO nematodes caused significantly more mortality than occurred in the control group (10 nematodes per cm^2^, ${\chi }_{3}^{2}=60.1$, *p* < 0.001; 25 nematodes per cm^2^, ${\chi }_{3}^{2}=51.7$, *p* < 0.001; 50 nematodes per cm^2^, ${\chi }_{3}^{2}=51.2$, *p* < 0.001). There was no difference in mortality between the different nematode concentration treatment groups. All concentrations of the VW nematodes also caused significantly more mortality than occurred in the control group (10 nematodes per cm^2^, ${\chi }_{3}^{2}=17.5$, *p* < 0.001; 25 nematodes per cm^2^, ${\chi }_{3}^{2}=34.6$, *p* < 0.001; 50 nematodes per cm^2^, ${\chi }_{3}^{2}=51.3$, *p* < 0.001). In this case mortality was affected by nematode concentration: exposure to 50 nematodes per cm^2^ caused higher mortality than exposure to 10 nematodes per cm^2^ (${\chi }_{3}^{2}=12.4$, *p* < 0.001).

### Nematode proliferation in infected bee carcasses

There was a notable difference in the ability of the two different entomopathogenic nematode products to proliferate in the carcasses of infected bees. *B. terrestris* proved a viable host for the entomopathogenic nematode species contained in the GYO product, and every nematode exposed bee carcass investigated had allowed a substantial number of infective juvenile nematodes to develop (see Table 1). There was no difference in nematode proliferation between the different nematode concentration treatment groups for the GYO product (${\chi }_{2}^{2}=3.26$, *p* = 0.196). In contrast to this, *B. terrestris* does not appear to be a viable host for the proliferation of *S. kraussei*, the infective agent in the VW product. Only 6 out of 48 bees which had died during the nematode exposure treatment yielded any infective juvenile nematodes at all, too few to compare nematode yield between different treatment groups. The control bee carcasses from both experiments which had died incidentally or been euthanised at the end of the study yielded no nematodes (*n* = 20), suggesting that the *B. terrestris* used in this study did not naturally host any nematode parasites.

**Table 1:** Nematode proliferation in the carcasses of bees which died during the nematode exposure experiment.

Nematode product bees were exposed to	Nematode exposure (nematodes per cm^2^ soil)	Number of carcasses studied	Number of carcasses with nematodes	Median number of nematodes per carcass	Lowest number recovered	Highest number recovered
Control	0	10	0	0	0	0
GYO	10	12	12	9,750	1,950	18,750
GYO	25	17	17	7,200	2,700	12,500
GYO	50	16	16	7,750	4,800	57,750
Control	0	10	0	0	0	0
VW	10	13	2	0	0	53,760
VW	25	17	3	0	0	6,800
VW	50	18	1	0	0	3,600

## Discussion

The results we present here show that the native British bumble bee *B. terrestris* is remarkably susceptible to two commercially available entomopathogenic nematode pest control products. Both products caused very high levels of bee mortality after only 72 h of exposure, with the first deaths evident after 48 h. This result was unexpected: the susceptibility of bees to entomopathogenic nematodes such as *Heterorhabdidtis spp*. and *Steinernema spp*. has never previously been reported.

The differences in nematode proliferation within the infected bee carcasses were also notable, with *Steinernema kraussei* failing to proliferate well in bee carcasses, while the product containing a mixture of *Heterorhabditis* and *Steinernema* species resulted in thousands of infective juveniles emerging from the bumble bee host. This may suggest that *Heterorhabditis* was the primary nematode proliferating within the bee carcasses, and the size of the nematodes recovered would to some extent support this; however without definitive genetic tests to determine the species recovered, and indeed the exact species mix within the GYO product, this remains speculative. Future studies using pure *Heterorhabditis* cultures would certainly help to confirm or refute this preliminary observation. It is possible that the bacterial symbiont of *Steinernema* species, *Xenorhabdus*, is able to overcome the bee immune defences and cause septicaemia, but is unable to sufficiently break down the tissues to allow nematode feeding and proliferation; whereas *Photorhabdus*, the bacterial symbiont of *Heterorhabditis*, may be able to digest the bee tissues more effectively and provide a better supply of nutrients to the nematodes (Nielsen-LeRoux et al., 2012). Alternatively, it is possible that as a parasite strain selected and maintained by the manufacturer for its pathogenicity of weevils, this particular isolate of *S. karussei* was less able to proliferate in a hymenopteran host.

This difference in proliferation, if confirmed in a field setting, has obvious implications for how ecologically significant the susceptibility of *B. terrestris* to entomopathogenic nematodes may be. Exposure to *S. kraussei* may kill individuals, but without any proliferation and release of infective juveniles then this species of nematode would only impact bees directly in contact with the initial application. However, the implications of entomopathogenic nematode sold as crop protection products being able to proliferate within *B. terrestris* as a viable host suggests this poses a greater risk of whole colonies becoming infected after a single individual exposure.

The fossorial habits of *B. terrestris*, and the overwintering of queens underground, may make this species uniquely vulnerable to biological pest control agents applied directly to the soil. Evidence from studies on the relative suspeptibility of pest species to entomopathogenic nematodes have shown that the lepidopteran *Cydia latiferreana*, a pest of hazelnuts which overwinters in soild or leaf litter as a pupa, is far more susceptible to nematode infection than a weevil pest of the same crop which has little contact with the soil (Chambers et al., 2010).

Although we acknowledge that this is a laboratory study and may not be directly applicable to a field setting, it highlights the need for further research into the off-target effects of biological pest control agents on insect pollinators. The conclusions of scientists advising the European regulatory body OECD in 1996 were that entomopathogenic nematodes were safe for wildlife and should not be regulated (Ehlers & Hokkanen, 1996). However, the main justification for a lack of regulation appears to be based on the premise that as multicellular animals, nematodes could not be regulated in the same way as entomopathogenic bacterial products such as *Bacillus thuringiensis*, and should instead only be regulated as introduced species if they are applied outside their country of origin (Ehlers & Hokkanen, 1996). The ideas underpinning the definition of entomopathogenic nematodes being safe for wildlife included the following: they pose no threat to mammals and birds (Boemare, Laumond & Mauleon, 1996); they have minimal adverse effects on above ground non-target invertebrates; (Akhurst, 1990) and they do not disperse widely in the environment (Downes & Griffin, 1996). Evidence of the effects of entomopathogenic nematodes applied directly to soil on non-target soil-dwelling invertebrates is lacking, and does not seem to have been included in these safety assessments.

Compared with other pathogen types, nematodes able to parasitise bees do not appear to be very diverse, or very well studied. *Bombus spp*. may be naturally infected by the parasitic nematode *Sphaerularia bombi*, which parasitises hibernating queens (Rutrecht & Brown, 2008), but a search of the literature reveals no other parasitic nematodes associated with bumblebees. Certainly, there is little evidence yet that nematode parasites used in pest control are infecting bumble bees in the field; but then it would appear that very few studies have been undertaken which would reveal the extent of such a phenomenon. What is evident from the literature is that several other introduced pathogens and parasites do in fact pose a serious threat to wild bumblebees: parasitic mites, *Nosema spp., Crithidia bombi*, neogregarine parasites, and several viruses including deformed wing virus, are all pathogens which are believed to have spread to wild bees from commercial bee rearing activities (Meeus et al., 2011). A degree of caution may therefore be advisable when deliberately introducing insect pathogens to the environment as biological pest control agents, until more is known about their effects on beneficial arthropods.

## Supplemental Information

10.7717/peerj.1413/supp-1 Data S1 Dataset from the study The file contains the raw data for bee mortality during exposure to entomopathogenic nematodes, as required by the journal.
